# Systematic identification of cancer pathways and potential drugs for intervention through multi-omics analysis

**DOI:** 10.1038/s41397-025-00361-6

**Published:** 2025-02-19

**Authors:** Tuan Xu, Deborah K. Ngan, Wei Zheng, Ruili Huang

**Affiliations:** Division of Preclinical Innovation, National Center for Advancing Translational Sciences (NCATS), National Institutes of Health (NIH), Rockville, MD 20850 USA

**Keywords:** Oncogenes, Drug development, Target identification

## Abstract

The pathogenesis of cancer is complicated, and different types of cancer often exhibit different gene mutations resulting in different omics profiles. The purpose of this study was to systematically identify cancer-specific biological pathways and potential cancer-targeting drugs. We collectively analyzed the transcriptomics and proteomics data from 16 common types of human cancer to study the mechanism of carcinogenesis and seek potential treatment. Statistical approaches were applied to identify significant molecular targets and pathways related to each cancer type. Potential anti-cancer drugs were subsequently retrieved that can target these pathways. The number of significant pathways linked to each cancer type ranged from four (stomach cancer) to 112 (acute myeloid leukemia), and the number of therapeutic drugs that can target these cancer related pathways, ranged from one (ovarian cancer) to 97 (acute myeloid leukemia and non-small-cell lung carcinoma). As a validation of our method, some of these drugs are FDA approved therapies for their corresponding cancer type. Our findings provide a rich source of testable hypotheses that can be applied to deconvolute the complex underlying mechanisms of human cancer and used to prioritize and repurpose drugs as anti-cancer therapies.

## Introduction

Cancer is a family of highly diverse and complex diseases that can occur in almost all organs and tissues of the human body. The occurrence and development of human cancers are associated with many factors, particularly the step-wise accumulation of genetic and epigenetic changes in the genome, which are directly manifested as alterations in the transcript and protein expression profiles [1]. High-throughput omics technologies (e.g., transcriptomics and proteomics) have been applied to identify potential biomarkers and novel therapeutic targets for the diagnosis and treatment of human cancers [2–4]. In addition, an integrative analysis across multiple omics data is capable of generating valid and testable hypotheses that can be prioritized for experimental validations [5]. Generally, the omics profiles vary with different types of cancer, and cancer research has focused primarily on various oncogenic processes associated with a specific cancer type. However, there are limited integrative multi-omics analyses across different cancer types that may reveal new pathways of cancer genesis and new therapeutic targets.

Cancer cell lines have been widely used as in vitro models for the investigation of the cellular and molecular mechanisms underlying tumorigenesis, as well as anti-cancer drug screening and repurposing [3, 4]. The Cancer Cell Line Encyclopedia (CCLE) is a publicly available database that contains multi-level omics data of over 1000 cancer cell lines spanning more than 40 cancer types. It provides RNA sequencing (RNA-Seq) transcriptomics data that measures RNA transcript abundance in the cancer cell lines [6]. In addition, the tandem mass tag (TMT) based quantitative proteomics approach has been used for large-scale protein quantification. Using this method, Nusinow et al. performed quantitative proteomics analysis on 375 cell lines across diverse cancer types, resulting in a rich resource of protein expression levels for the exploration of cellular behavior and cancer research [2]. Transcriptomics and proteomics play pivotal roles in linking genomic transcript sequences and protein levels to potential biological functions. Therefore, integrating these two omics methods (i.e., transcriptomics and proteomics) can provide a more comprehensive and holistic understanding of the biological behaviors of cancer at the transcriptional and translational levels that may reveal new mechanisms of pathogenesis and drug targets for cancer.

Understanding molecular targets characteristic of a cancer type is crucial for modern anti-cancer drug discovery and therapeutic development. For example, discoidin domain receptor 1 (DDR1) was identified as a molecular target specific for pancreatic cancer. This discovery enabled the development of a novel series of 2-amino-2,3-dihydro-1H-indene-5-carboxamide derivatives as highly selective DDR1 inhibitors using structure-based drug design. These DDR1 inhibitors showed promising efficacy for pancreatic cancer treatment [7]. Omics analysis, either RNA-Seq or proteomics profiling, has provided a rapidly expanding range of information on new molecular targets for early drug discovery. For example, Swaroop et al. found that the genes differentially expressed in the most severe Hurler syndrome subgroup compared to the intermediate Hurler-Scheie or the least severe Scheie syndrome subgroups based on transcriptome profiling data were extremely valuable in guiding the in vivo animal models and clinical trials in the drug development process [8].

In this study, we integrated the transcriptomics and proteomics data from 16 common human cancer types, including acute myeloid leukemia (AML), breast cancer, colorectal cancer, endometrial cancer, esophageal cancer, glioma, kidney cancer, liver cancer, non-small-cell lung carcinoma (NSCLC), small cell lung carcinoma (SCLC), melanoma, ovarian cancer, pancreatic cancer, stomach cancer, upper aerodigestive cancer, and urinary tract cancer, to identify the biological pathways characteristic of each cancer type and drugs known to target these pathways. The cancer pathways identified in this study can provide insight into the underlying molecular mechanisms for each cancer type, and the drugs targeting these pathways could potentially be repurposed as new cancer therapeutics.

## Results

### Overview of cancer profiling data

**Fig. 1:**
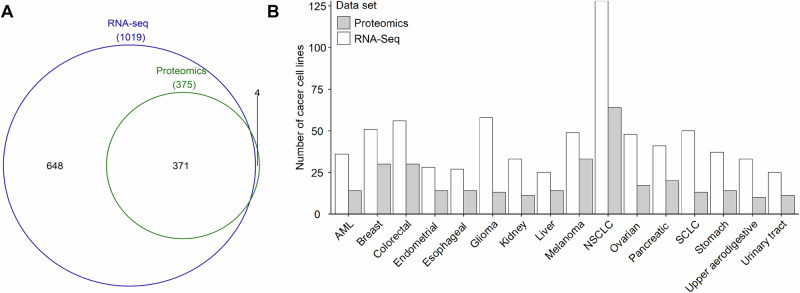
Overview of cancer types. **A** The number of cancer cell lines with RNA-Seq data and proteomics data. **B** The distribution of cell lines for each cancer type. The RNA-Seq data were obtained from the Cancer Cell Line Encyclopedia (CCLE) database [6], and the proteomics data were derived from the peer-reviewed published literature [2]. The cancer type was defined according to the cancer cell line annotations from the CCLE database.

A total of 1023 human cancer cell lines were collected, including 1019 cell lines with RNA-Seq data and 375 cell lines with proteomics data (Fig. 1A, and Supplementary Table S1). Of the cancer cell lines collected, 371 had both RNA-Seq and proteomics data (Fig. 1A, and Supplementary Table S1). The four cell lines that had only proteomics data were COLO205 (large intestine cancer), PL45 (pancreatic cancer), SKMEL2 (skin cancer), and NB19 (central nervous system cancer) (Supplementary Table S1). According to the cancer cell line annotations, these cancer cell lines can be grouped into 16 cancer types, including AML, breast cancer, colorectal cancer, endometrial cancer, esophageal cancer, glioma, kidney cancer, liver cancer, NSCLC, SCLC, melanoma, ovarian cancer, pancreatic cancer, stomach cancer, upper aerodigestive cancer, and urinary tract cancer (Fig. 1B, and Supplementary Table S1). The number of cancer cell lines with proteomics data for each cancer type was significantly smaller than those with RNA-Seq data (Fig. 1B). For cancer types with RNA-Seq data, the number of cancer cell lines ranged from 25 (liver cancer and urinary tract cancer) to 128 (NSCLC) with a median of 41 (Fig. 1B, and Supplementary Table S1). For cancer types with proteomics data, the number of cell lines ranged from 10 (upper aerodigestive cancer) to 64 (NSCLC) with a median of 14 (Fig. 1B, and Supplementary Table S1).

### Transcripts and proteins significantly expressed in each cancer type

**Fig. 2:**
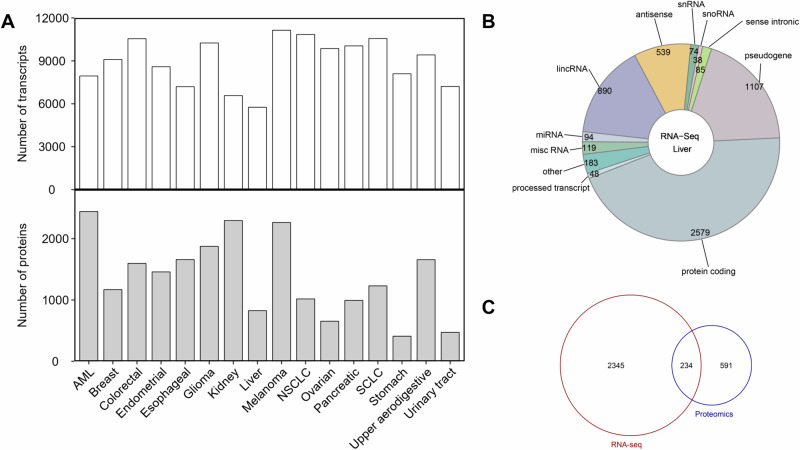
Significant transcripts and proteins identified for each cancer type. **A** The distribution of the significant transcripts and proteins. **B** The composition of the transcripts in liver cancer. **C** The overlapping of the significant transcripts and proteins associated with liver cancer. The statistical comparison between a specific cancer type and the others was initially performed to calculate a *P* value using the unpaired two-tailed Student’s *t* test. The significant transcripts of each cancer type were prioritized through a combination of smaller FDR-adjusted *P* value and higher Gini purity. In contrast to transcript, a *P* value of <0.05 was defined as the selection criteria for the significant proteins of each cancer type.

According to the optimal combination of Gini purity and FDR adjusted *P* value, the number of significant transcripts for each cancer type ranged from 5756 (liver cancer) to 11,143 (melanoma) with a median of 9256 (Fig. 2A, and Supplementary Table S2). Transcripts that showed statistically significant differential expression in a specific cancer type compared to all other cancer types are referred to as “significant transcripts” here. The number of significant proteins for each cancer type ranged from 409 (stomach cancer) to 2443 (AML) with a median of 1344 (Fig. 2A). The number of significant proteins is much smaller than that of the significant transcripts for each cancer type, and the transcript/protein ratio ranged from 2.86 (kidney cancer) to 19.8 (stomach cancer) with a median of 6.79 (Fig. 2A). Transcript is a collective term that includes various biotypes. For example, among the 5756 significant transcripts found for liver cancer included 23 biotypes, the top 10 biotypes in descending number of transcripts were protein coding (2579), pseudogene (1107), lincRNA (890), antisense (539), misc RNA (119), miRNA (94), sense intronic (85), snRNA (74), processed transcript (48), and snoRNA (38), accounting for 96.8% of all the biotypes (Fig. 2B). Moreover, 234 protein coding biotypes in the significant transcript set (a total of 2579 transcripts) were also present in the significant protein set (a total of 825 proteins) for liver cancer (Fig. 2C), showing that the results from the transcriptomics analysis and the proteomics analysis are consistent. These significantly expressed transcripts and proteins are specific for a particular cancer type and can be used for cancer type-specific pathway analysis.

### Biological pathways characteristic of each cancer type

**Fig. 3:**
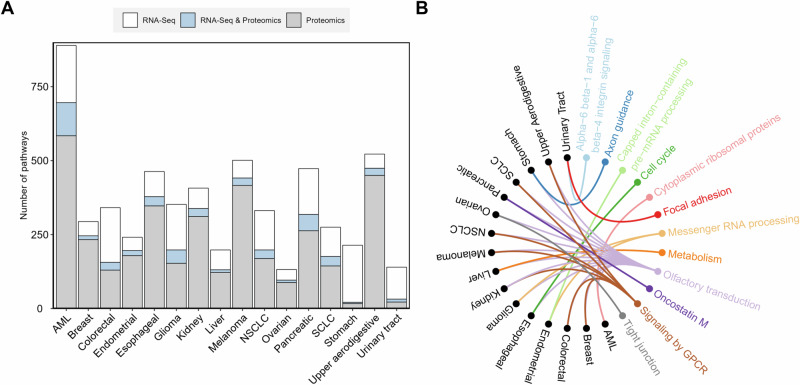
Biological pathways found significantly associated with each cancer type. **A** Distribution of the significant pathways. **B** The most significant biological pathway for each cancer type. The 16 different cancer types are shown in black, and the significant biological pathways are shown in colored fonts. The colored connecting lines represent associations found between cancer types and pathways. The significant pathways for each cancer type were identified using the Fisher’s exact test with FDR and bootstrap. The significant biological pathways for each cancer type were ranked and prioritized by their combined significance scores (e.g., a smaller score indicates a more significant relationship between the pathway and the cancer type), which was the average of the logarithms of the FDR adjusted *P* values from the transcript and protein analysis results. The enriched pathways were identified based on the NCATS BioPlanet database, which currently aggregates 2270 human biological pathways from multiple publicly available sources [56].

The significant transcripts and proteins for each cancer type were analyzed for the enrichment of biological pathways, respectively. From the significant transcripts, the number of significant pathways ranged from 36 (ovarian cancer) to 193 (AML and stomach cancer) with a median of 92 (Fig. 3A). From the significant proteins, the number of significant pathways ranged from 17 (stomach cancer) to 584 (AML) with a median of 174 (Fig. 3A). The number of overlapping pathways derived from both transcripts and proteins for each cancer type ranged from 4 (stomach cancer) to 112 (AML) with a median of 25.5 (Fig. 3A, and Supplementary Table S3). The overlapping significant pathways were considered characteristic of each cancer type. Some pathways were present in multiple cancer types, while others were specific for a particular cancer type (Supplementary Table S3). Figure 3B showed the top two significant pathways found for each cancer type, including 12 unique biological pathways. For example, the olfactory transduction pathway was the significant pathway for AML (score = −20.52), breast cancer (score = −14.18), colorectal cancer (score = −21.92), esophageal cancer (score = −8.37), glioma (score = −22.90), kidney cancer (score = −15.21), liver cancer (score = −4.64), melanoma (score = −26.34), NSCLC (score = −22.46), ovarian cancer (score = −6.32), pancreatic cancer (score = −7.17), SCLC (score = −22.44), stomach cancer (score = −4.97), and upper aerodigestive cancer (score = −25.98). Signaling by the GPCR pathway was the significant pathway for breast cancer (score = −5.56), colorectal cancer (score = −10.42), kidney cancer (score = −16.22), melanoma (score = −14.41), NSCLC (score = −7.91), SCLC (score = −10.81), and upper aerodigestive cancer (score = −14.44). Messenger RNA processing was the significant pathway for endometrial cancer (score = −19.50) and glioma (score = −14.69). Alpha-6 beta-1 and alpha-6 beta-4 integrin signaling pathway was the significant pathway for urinary tract cancer (score = −3.84). Axon guidance pathway was the significant pathway for stomach cancer (score = −4.15). Capped intron-containing pre-mRNA processing pathway was the significant pathway for endometrial cancer (score = −18.58). Cell cycle pathway was the significant pathway for esophageal cancer (score = −4.15). Cytoplasmic ribosomal proteins pathway was the significant pathway for AML (score = −11.46). Focal adhesion pathway was the significant pathway for urinary tract cancer (score = −3.39). Metabolism pathway was the significant pathway for liver cancer (score = −3.22). Oncostatin M pathway was the significant pathway for pancreatic cancer (score = −6.08). Tight junction pathway was the significant pathway for ovarian cancer (score = −2.09).

### Potential anti-cancer drugs identified for each cancer type

**Fig. 4:**
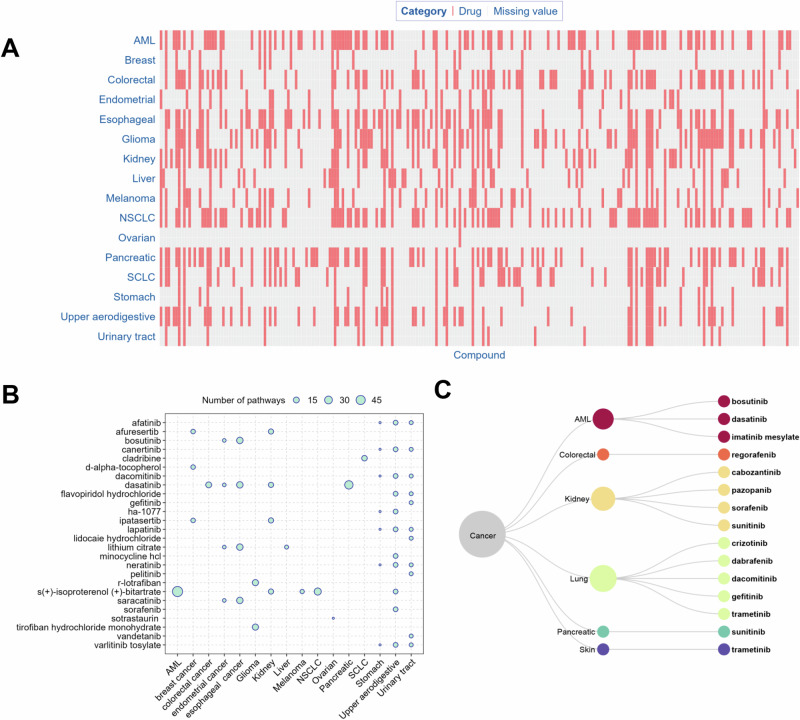
Anti-cancer drugs as potential treatment for each cancer type. **A** Distribution of the potential anti-cancer drugs. The term “missing value” indicates that the respective compound is not regarded as a potential treatment for the specific cancer in our study. **B** Drugs involved in the maximal number of significant pathways. The circles represent the number of pathways linking a drug and a cancer type. For each cancer type, only the drugs that share the largest number of pathways with it are shown in the figure. **C** FDA approved, targeted anti-cancer drugs and their corresponding cancer types. The drug-molecular target annotations were downloaded from the DrugBank and KEGG drug databases [58]. The FDA approved anti-cancer drugs were derived from the NCI website (https://www.cancer.gov/about-cancer/treatment/types/targeted-therapies/targeted-therapies-fact-sheet).

The significant cancer pathways can serve as a bridge connecting drugs and cancer type. For each cancer type, we identified the drugs that target genes involved in multiple significant cancer pathways. In turn, these drugs can serve as potential anti-cancer drug candidates. The number of potential anti-cancer drugs varied by cancer types, ranging from 1 (ovarian cancer) to 97 (AML and NSCLC) with a median of 66 (Fig. 4A, Supplementary Table S4). For each cancer type, the drugs linked to the maximal number of pathways are shown in Fig. 4B and Supplementary Table S4, and these drugs can be divided into two categories: those involved with multiple cancer types and those involved with one specific cancer type. The former included S(+)-isoproterenol bitartrate for AML (58 pathways), kidney cancer (11 pathways), NSCLC (24 pathways), melanoma (7 pathways), and upper aerodigestive (9 pathways); afatinib for stomach cancer (3 pathways) and upper aerodigestive cancer (9 pathways); afuresertib for breast cancer (8 pathways) and kidney cancer (11 pathways); bosutinib for endometrial cancer (5 pathways) and esophageal cancer (19 pathways); canertinib for stomach cancer (3 pathways) and upper aerodigestive cancer (9 pathways); dacomitinib for stomach cancer (3 pathways) and upper aerodigestive cancer (9 pathways); dasatinib for colorectal cancer (15 pathways), endometrial cancer (5 pathways), esophageal cancer (19 pathways), kidney cancer (11 pathways), and pancreatic cancer (36 pathways); HA-1077 for stomach cancer (3 pathways) and upper aerodigestive cancer (9 pathways); ipatasertib for breast cancer (8 pathways) and kidney cancer (11 pathways); lithium citrate for endometrial cancer (5 pathways), esophageal cancer (19 pathways), and liver cancer (6 pathways); neratinib for stomach cancer (3 pathways) and upper aerodigestive cancer (9 pathways); saracatinib for endometrial cancer (5 pathways) and esophageal cancer (19 pathways); varlitinib tosylate for stomach cancer (3 pathways) and upper aerodigestive cancer (9 pathways). The latter included flavopiridol hydrochloride (9 pathways), lapatinib (9 pathways), minocycline HCl (9 pathways), and sorafenib (9 pathways) for upper aerodigestive; cladribine (13 pathways) for SCLC; D-alpha-tocopherol (8 pathways) for breast cancer; lapatinib (3 pathways) for stomach cancer; R-lotrafiban (17 pathways) and tirofiban hydrochloride monohydrate (17 pathways) for glioma; and sotrastaurin (3 pathways) for ovarian cancer (Fig. 4B, and Supplementary Table S4). Some anti-cancer drugs identified in this study have been approved as targeted therapies for the treatment of specific cancer types (Fig. 4C), such as imatinib, bosutinib, and dasatinib for AML; dabrafenib, crizotinib, trametinib, dacomitinib, and gefitinib for lung cancer; regorafenib for colorectal cancer; pazopanib, cabozantinib, sunitinib malate, and sorafenib for kidney cancer; trametinib for skin cancer; and sunitinib malate for pancreatic cancer.

### Quantitative validation by mean normalized AUC (mnAUC)

A total of 426 potential anti-cancer drugs (~44% of the total) were identified, with mnAUC values ranging from 0.23 to 1.42 and a median of 0.88 (Table S4). The number of anti-cancer drugs with available mnAUC values varied across cancer types: breast (7), stomach (17), endometrium (24), liver (25), SCLC (36), colorectal (40), kidney (45), pancreas (48), glioma (49), esophagus (52), and NSCLC (62). A Wilcoxon rank-sum test revealed that the mean mnAUC value (0.87) of potential anti-cancer drugs identified in this study was significantly lower than that (0.96) for 19,759 potential anti-cancer drugs reported in the literature (*p* < 2 × 10^–16^; Fig. S1).

## Discussion

In this study, we identified the transcripts and proteins significantly expressed in each of the 16 cancer types through integrated analysis of transcriptomics and proteomics profiling data, resulting in biological pathways characteristic of each cancer type. Moreover, the drugs linked to these biological pathways were identified as potential treatments for human cancer.

According to the global cancer statistics in 2020, the cancer types analyzed in our study (Fig. 1, and Supplementary Table S1) included the most commonly diagnosed cancer (breast cancer, 11.7% of all sites) and the cancer with the leading death rate (lung cancer, 18% of all sites) [9]. As proteins are the key executors of gene function, high-throughput proteomics data are important in elucidating the mechanisms of action of many critical cancer-related biological processes [2]. Due to the constrained resolution at the proteome level, the coverage of proteomics data is much lower than that of RNA-Seq data, resulting in a smaller number of significant proteins comparing to the number of significant transcripts for each cancer type identified in our study (Fig. 2A). The protein levels in cells may not correlate with the expression levels of transcripts because of an underlying epigenetic mechanism [10]. In addition to protein-encoding mRNAs, the transcripts also included non-coding RNAs (e.g., long non-coding RNA (lncRNA) and microRNA (miRNA)), some of which often act as oncogenic drivers and tumor suppressors in major cancer types through post-transcriptional regulatory mechanisms [11–13].

We also identified the significant pathways characteristic of each cancer type (Fig. 3, and Supplementary Table S3), some of which have been reported to  be associated with the corresponding human cancer type. For example, the olfactory transduction pathway has been reported to be associated with certain cancer types including breast cancer [14], pancreatic cancer [15], lung carcinoids [16], colorectal cancer [17], ovarian serous cystadenocarcinoma [18], stomach cancer [18], esophageal cancer [18], and brain lower grade glioma [18]. Furthermore, the olfactory receptor (OR) family is generally considered to play an important role in the olfactory transduction pathway and a link to various cancers, such as human melanoma, stomach cancer, and AML [19, 20]. In our study, the olfactory transduction pathway has been identified as significant for 16 cancer types (i.e., AML, breast cancer, colorectal cancer, endometrial cancer, esophageal cancer, glioma, kidney cancer, liver cancer, NSCLC, SCLC, melanoma, ovarian cancer, pancreatic cancer, stomach cancer, upper aerodigestive cancer, and urinary tract cancer) (Fig. 3, and Supplementary Table S3). The axon guidance pathway has reported cancer associations, e.g., the axon guidance factor Slit homolog 2 (Slit 2) is known to inhibit neural invasion and metastasis in pancreatic cancer [21], and affect the prognosis of AML [22]. Silencing of the axon guidance factor semaphorin 6B gene significantly suppressed adhesion, migration, and invasion of stomach cancer cells in vitro [23]. Consistent with these previous studies, the axon guidance pathway was also found closely related to pancreatic cancer, AML, and stomach cancer in our study (Fig. 3, and Supplementary Table S3). Guanine nucleotide-binding protein (G protein) coupled receptors (GPCRs) are the largest family of membrane receptors that mediate transmembrane signaling via heterotrimeric G protein complexes. GPCR signaling has been implicated in various oncogenic and metastatic processes [24, 25]. Consistent with these previous studies, the GPCR signaling pathway was also found closely related to AML, breast cancer, colorectal cancer, glioma, kidney cancer, NSCLC, SCLC, melanoma, ovarian cancer, and upper aerodigestive cancer in our study (Fig. 3, and Supplementary Table S3).

These cancer pathways also led to the identification of existing drugs that could potentially be repurposed as new anti-cancer therapies (Fig. 4, and Supplementary Table S4). Drugs that target multiple biological pathways simultaneously may produce additive or even synergistic anti-cancer effects, resulting in more effective therapies and reduced side effects [26, 27]. Figure 4B shows the drugs that are linked to the maximum number of pathways for each cancer type. For example, dasatinib, a small molecule tyrosine kinase inhibitor, has been found to inhibit the growth of AML, breast cancer, liver cancer, melanoma, pancreas tumor, and pre-neoplastic Barrett’s esophagus cell lines [28–34]. Although dasatinib has previously been reported to inhibit the growth of NSCLC but not SCLC [35], recent studies have found that dasatinib can significantly enhance the therapeutic efficacy of vorinostat in SCLC xenografts [36]. In addition, dasatinib has been reported to induce autophagic cell death in human ovarian cancer [37]. Consistent with these previous studies, we found dasatinib among the drug candidates for AML, breast cancer, colorectal cancer, endometrium cancer, esophageal cancer, glioma, kidney cancer, liver cancer, melanoma, pancreatic cancer, NSCLC, upper aerodigestive cancer, urinary tract cancer, and SCLC (Fig. 4B, and Supplementary Table S4). Afuresertib is a potent protein kinase B (AKT) inhibitor that exhibits favorable tumor-suppressive effects on breast cancer cells by potently inhibiting the phosphatidylinositol 3‑kinase (PI3K)/AKT signaling pathway [38]. Consistent with this study, Afuresertib is one of the drugs we found linked to breast cancer (Fig. 4B, and Supplementary Table S4). D-alpha-tocopherol plays a pivotal role in decreasing the metastasis risk of glioma in cancer patients [39]. We also found D-alpha-tocopherol as one of the drugs linked to glioma (Supplementary Table S4). Ipatasertib is a potent small molecule AKT kinase inhibitor currently being tested in Phase III clinical trials for the treatment of triple negative metastatic breast cancer [40], which is also linked to breast cancer in our study (Fig. 4B, and Supplementary Table S4). Consistent with the linkage of midostaurin to glioma by our analysis (Supplementary Table S4), midostaurin is a multi-targeted tyrosine kinase inhibitor for the treatment of glioma [41]. In addition to these drugs with confirmed anti-cancer activity in the literature, the other drugs identified in our study could potentially be prioritized and repurposed as new treatments for some cancer types. For example, the Rho-kinase inhibitor, HA-1077, suppresses proliferation/migration and induces apoptosis of urothelial cancer cells [42] and MDA-MB 231 human breast cancer cells [43], while our analysis additionally linked HA-1077 to colorectal cancer and stomach cancer (Fig. 4B, and Supplementary Table S4).

Moreover, some potential anti-cancer drugs identified in our study have been screened for anti-cancer activities in cell-based assays. For example, dasatinib was associated with 16 significant pathways for colorectal cancer (Supplementary Table S4), and inhibited the viability of colorectal cancer cells in vitro (i.e., IC_50_ = 0.40 μM, efficacy = 57%) [44]. Enzastaurin was associated with five significant colorectal cancer pathways (Supplementary Table S4), and inhibited colorectal cancer cell viability in vitro (i.e., IC_50_ = 11 μM, efficacy = 54%) [44]. Finally, puromycin, a drug linked to four significant glioma pathways in our study (Supplementary Table S4), was also found to reduce the viability of glioblastoma cells in vitro (i.e., IC_50_ = 2.74 μM, efficacy = 90%) [45]. In addition, some drugs identified by our approach are approved targeted therapies for their corresponding cancer type. These findings provide additional evidence for the utility of our method (Fig. 4C). The Profiling Relative Inhibition Simultaneously in Mixtures (PRISM) repurposing dataset provides information on the growth inhibitory activity of 4518 drugs tested across 578 human cancer cell lines, and the area under the dose-response curve (AUC) is a metric that represents the fraction of cells left after drug exposure averaged over all the test concentrations normalized to cells receiving no drug treatment [46]. Given the variability in cell line testing across different drugs in the PRISM dataset, Koudijs et al. utilized a linear mixed model to separate the effect of cell lines and drugs. They then consolidated the findings into estimating the mean normalized AUC (mnAUC) that represents the average fraction of cells left after drug exposure in a group of cell lines [47]. In this study, mnAUC values for the identified potential anti-cancer drugs were calculated using the methodology of Koudijs et al. to assess drug efficacy (Table S4). A Wilcoxon rank-sum test revealed that the mnAUC values of the anti-cancer drugs identified in this study were significantly lower than those reported for potential anti-cancer drugs in the literature (*p* < 2 × 10^−16^), indicating that the identified drugs demonstrated robust anti-cancer effects against their respective cancer types (Figure S1). To evaluate the efficacy of the method in identifying drugs for specific cancer types, a randomization test was conducted to compare hit rates between our method and the randomized selections. A drug-cancer type pair was defined as a hit if the drug is an approved targeted therapy for the corresponding cancer type. In the randomization test, 1000 cancer type-drug pairs were sampled from the raw data 100 times, yielding an average hit rate of 0.2%, which was significantly lower than the hit rate of 1.5% for the 974 pairs (Fisher’s exact test, *p* = 0.001) predicted by our method in this study.

In this study, we employed an integrated multi-omics approach, which has demonstrated numerous advantages over conventional single-omics methods. For example, Deng et al. utilized an integrated approach by incorporating transcriptomic, proteomic, and metabolomic molecular profiles of tumor patients. This data integration strategy facilitated the identification of key pathways and metabolites, surpassing the accuracy achieved by individual transcriptomic analyses [48]. Similarly, Lu et al. conducted a thorough analysis by integrating transcriptomic and proteomic data in glioblastoma. The results revealed a significant enrichment of the gonadotropin-releasing hormone (GnRH) signaling pathway, a finding not discernible through single omics datasets. This highlights the potential of multi-omics research and analyses in providing a more comprehensive understanding of complex cancers [49]. Furthermore, Heo et al. found that the integration of multi-omics data offers a comprehensive depiction of the molecular and clinical profile of cancer patients when contrasted with single-omics approaches. This integration not only enhanced the generation of high-quality, unbiased datasets, but also contributed to a more holistic understanding of the subject [50]. Our study is one of many that have utilized the CCLE database in different ways to achieve various goals in cancer research and drug discovery. For example, Shao et al. employed a recommendation system learning model with CCLE data (i.e., drug data and multi-omics data in CCLE), focusing on drug-drug functional similarities, unlike our study, which identified cancer type-specific drugs [51]. Hsu et al. developed Scaden-CA, a deep learning model for deconvoluting tumor data into proportions of cancer type-specific cell lines, aiming to bridge the gap in pharmacogenomics knowledge between in vitro and in vivo datasets. The CCLE bulk RNA data was used for their model validation [52]. Carvalho et al. used CCLE data (i.e., copy number and RNA-Seq expression data of colorectal cancer cell lines in CCLE) to identify cell line models and explore drug responses in rectal cancer, revealing significant findings related to the topoisomerase 2A (TOP2A) gene in separate patient cohorts [53]. Mohammadi et al. analyzed proteomics data from 26 breast cancer cell lines in the CCLE to examine the expression patterns of specific antimicrobial and immunomodulatory peptides across various breast cancer subtypes, aiming to facilitate drug repurposing efforts [54]. Rinaldetti et al. used transcriptome expression data from CCLE and BLA-40 cell lines to identify novel subtype-stratified therapeutic approaches for muscle-invasive bladder cancer through high-content screening, revealing distinct drug sensitivities and highlighting the role of CCLE in molecular subtype assignments [55].

## Conclusions

We performed an integrative analysis of large-scale RNA-Seq and proteomics profiling data, resulting in a set of characteristic pathways for 16 human cancer types. These pathways can provide a systematic understanding of the complex underlying mechanisms for each cancer type. Furthermore, through these characteristic cancer pathways, we identified drugs for each cancer type, which could serve as drug repurposing candidates for cancer treatment. Our results provide a rich set of testable hypotheses for the design of future experimental validation and clinical trials.

## Data and methods

### Data collection

RNA-Seq data (file: CCLE_RNAseq_genes_rpkm_20180929.gct) were retrieved from the CCLE database, and these data contain a total of 1019 cancer cell lines with 56,202 different transcripts [6]. Quantitative proteomics data were obtained from the literature, and these data contain a total of 375 cancer cell lines with 12,755 different proteins [2]. Cancer cell line annotations (file: Cell_lines_annotations_20181226.txt) were downloaded from the CCLE database [6]. To quantitatively validate the results, mean normalized Area Under the Curve (mnAUC) data were utilized from the supplementary materials of a previously published study [47]. The mnAUC values reflect the average fraction of surviving cells after drug exposure across multiple cell lines.

### Identification of significant transcripts and proteins for each cancer type

The raw transcriptome data were pre-processed to remove outliers using the capping method (i.e., the maximum RPKM value for each cell line was calibrated to the value that occurs most frequently among the maximum RPKM values for all cell lines), followed by a log2 transformation. The raw proteomics data were not subjected to the same preprocessing steps as the transcriptome data, as they had already undergone a log2 transformation. To identify the transcripts and proteins specific for each cancer type, we first determined if there was any significant difference between their expression levels across different cancer types using one-way analysis of variance (ANOVA). Transcripts or proteins that showed significant differential expression (*P* value < 0.05) were further analyzed to see if they were significantly expressed for a specific cancer type. The expression levels for one cancer type were compared with those of the others, and statistical significance was determined by the *P* value from a two-tailed Student’s *t* test. For each cancer type, the resulting *P* values were then corrected for multiple hypothesis testing using the false discovery rate (FDR), and the FDR-adjusted *P* values were set from 10^−10^ to 10^−2^ with a tenfold proportional increase. Each transcript subset at a different FDR-adjusted *P* value cutoff was subsequently clustered hierarchically using the complete linkage method with the Euclidean distance as the similarity metric. The clustering results were quantified using Gini purity, a measure of clustering specificity. The value of Gini purity ranged from 0 to 1, with higher values indicating higher specialization in the cluster. Finally, the significant transcripts for each cancer type were prioritized based on the FDR-adjusted *P* value and Gini purity. For protein expression data, a *P* value of <0.05 was used to select the significant proteins for each cancer type.

### Biological pathway enrichment analysis

The NCATS BioPlanet pathway database was used to identify the biological pathways characteristic of each cancer type [56]. The pathways enriched in each transcript or protein set for a particular cancer type was determined in two steps. The Fisher’s exact test was first applied and then the FDR was calculated. The statistical significance of the pathways with an FDR adjusted *P* value < 0.05 was further assessed via bootstrap with 1000 replications. The bootstrap *P* value was calculated by counting the number of times the Fisher’s exact *P* value from the randomly permutated data was smaller than the true observed value, i.e., a bootstrap *P* value of 0.005 means that five out of the 1000 random *P* values were smaller than the true observed *P* value. A bootstrap *P* value < 0.05 was considered statistically significant. To improve the reliability of the pathways identified, the enrichment *P* values from the transcripts and proteins were further combined into a significance score (i.e., the average of the logarithms of the FDR adjusted *P* values). The significant biological pathways for each cancer type were ranked and prioritized by this combined score (e.g., a smaller score indicates a higher level of significance).

### Identification of potential anti-cancer drugs

Drug target annotations were acquired from the DrugBank database (https://go.drugbank.com/) and the Kyoto Encyclopedia of Genes and Genomes (KEGG) drug database (https://www.genome.jp/kegg/drug/). DrugBank is a bioinformatics and cheminformatics resource that combines detailed drug data with comprehensive target information [57]. The KEGG drug database stores abundant information pertaining to drugs and their interacting molecular targets, which could be useful in the development of new potential anti-cancer drugs [58]. Anti-cancer drug candidates were identified based on the drug-target interactions annotated by the above two databases. Molecular targets involved in multiple biological pathways significant for a cancer type were collected for drug candidate identification. Approved targeted cancer therapies and their corresponding cancer types were retrieved from the National Cancer Institute (NCI) at the National Institutes of Health (NIH) website (https://www.cancer.gov/about-cancer/treatment/types/targeted-therapies/targeted-therapies-fact-sheet).

## Supplementary information

Table S1

Table S2

Table S3

Table S4

Supplementary file

## Data Availability

The complete code package and dataset can be accessed via a GitHub repository (https://github.com/TX-2017/multi-omics-analysis).

## Code availability

The complete code package and dataset can be accessed via a GitHub repository (https://github.com/TX-2017/multi-omics-analysis).
