# Supplementary material for: Systematic identification of cancer pathways and potential drugs for intervention through multi-omics analysis
Source: Pharmacogenomics J. 2025 Feb 19;25(1-2):2. doi: 10.1038/s41397-025-00361-6 (PMC11839471; doi:10.1038/s41397-025-00361-6)
Supplement: Supplementary file 5 — Supplementary file [file 41397_2025_361_MOESM5_ESM.docx]

Systematic identification of cancer pathways and potential drugs for intervention through multi-omics analysis

Tuan Xu, Deborah K. Ngan, Wei Zheng, Ruili Huang*

Division of Preclinical Innovation, National Center for Advancing Translational Sciences (NCATS), National Institutes of Health (NIH), Rockville, Maryland 20850, United States

* Correspondence:

Ruili Huang, Ph.D.

9800 Medical Center Drive

DPI/NCATS

National Institutes of Health

Rockville, MD 20850

Phone: 301-827-0944

Fax: 301-217-5736

Email: [huangru@mail.nih.gov](mailto:huangru@mail.nih.gov)

Figure S1


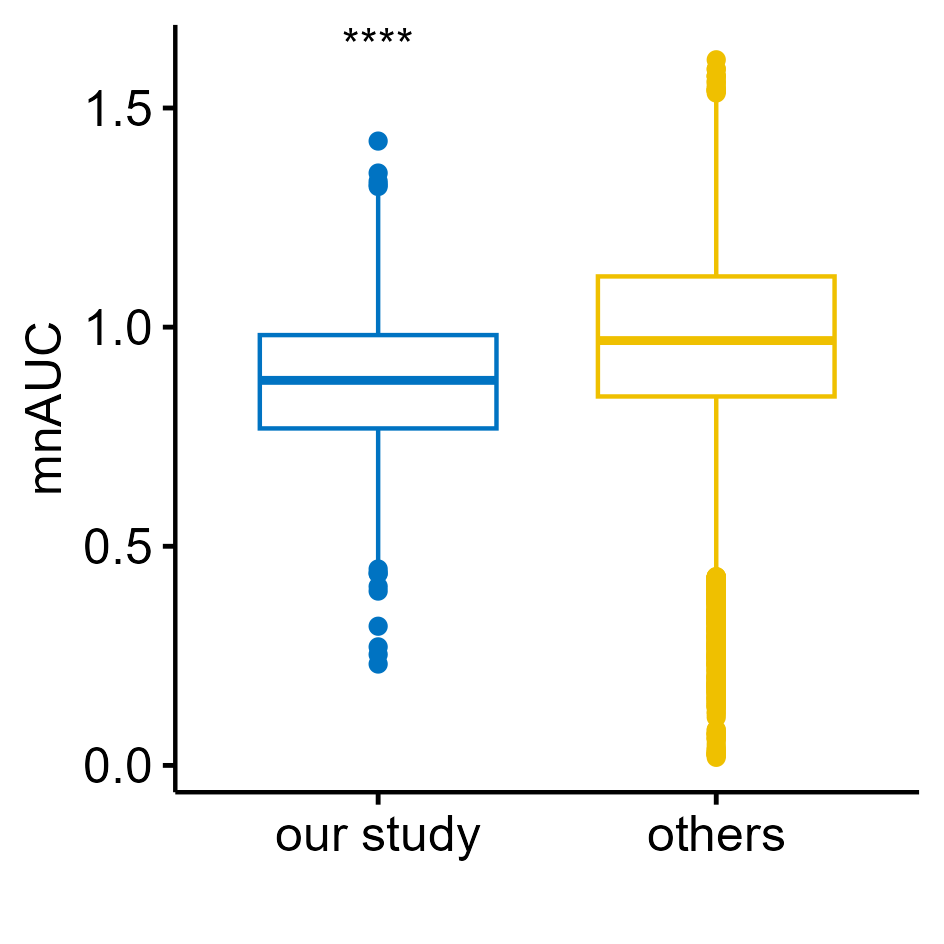


Figure S1. Comparison of mean normalized Area Under the Curve (mnAUC) values between the potential anti-cancer drugs identified in this study and anti-cancer drugs reported in the literature (others). P values were calculated using the Wilcoxon rank-sum test; **** *P* < 0.0001.
